# *L*-Tryptophan: Basic Metabolic Functions, Behavioral Research and Therapeutic Indications

**DOI:** 10.4137/ijtr.s2129

**Published:** 2009-03-23

**Authors:** Dawn M Richard, Michael A Dawes, Charles W Mathias, Ashley Acheson, Nathalie Hill-Kapturczak, Donald M Dougherty

**Affiliations:** 1 Neurobehavioral Research Laboratory and Clinic, Department of Psychiatry; 2 Research Imaging Center, University of Texas Health Science Center at San Antonio, U.S.A

**Keywords:** L-tryptophan, depletion, loading, therapeutics, clinical uses, metabolism

## Abstract

An essential component of the human diet, L-tryptophan is critical in a number of metabolic functions and has been widely used in numerous research and clinical trials. This review provides a brief overview of the role of L-tryptophan in protein synthesis and a number of other metabolic functions. With emphasis on L-tryptophan’s role in synthesis of brain serotonin, details are provided on the research uses of L-tryptophan, particularly L-tryptophan depletion, and on clinical trials that have been conducted using L-tryptophan supplementation. The ability to change the rates of serotonin synthesis in the brain by manipulating concentrations of serum tryptophan is the foundation of much research. As the sole precursor of serotonin, experimental research has shown that L-tryptophan’s role in brain serotonin synthesis is an important factor involved in mood, behavior, and cognition. Furthermore, clinical trials have provided some initial evidence of L-tryptophan’s efficacy for treatment of psychiatric disorders, particularly when used in combination with other therapeutic agents.

## Introduction

Hopkins and Cole^1^ discovered tryptophan in the early 1900s after isolating it from casein protein, and Ellinger and Flamand^2^ determined its molecular structure a short time later. *L*-tryptophan (i.e. tryptophan) is one of eight essential amino acids (i.e. amino acids that cannot be synthesized in the human body and must be supplied by the diet).^3^–^5^ For all amino acids, including *L*-tryptophan, only the *L* isomer is used in protein synthesis^6^ and can pass across the blood-brain barrier.^7^,^8^ In humans, tryptophan has relatively low tissue storage^9^ and the overall tryptophan concentration in the body is the lowest among all amino acids,^10^,^11^ although only small amounts are necessary for general healthy nutrition.^5^,^12^ While typical intake for many individuals is approximately 900 to 1000 mg daily, the recommended daily allowance for adults is estimated to be between 250 mg/day^5^,^12^,^13^ and 425 mg/day,^4^,^14^,^15^ which translates to a dietary intake of 3.5 to 6.0 mg/kg of body weight per day. Some common sources of tryptophan are oats, bananas, dried prunes, milk, tuna fish, cheese, bread, chicken, turkey, peanuts, and chocolate (see Table 1).^11^,^16^

Tryptophan was first synthesized in 1949, but by the early 1980’s chemical synthesis of tryptophan was replaced with fermentation procedures that greatly increased obtainable yields, making tryptophan supplements more available.^17^ Shortly thereafter, from approximately 1988 to 1989, there was an out-break of eosinophilia-myalgia syndrome (EMS) that was linked to consumption of synthetic tryptophan. Upon investigation, the source of the outbreak was traced to a single manufacturer, the Showa Denka Company of Japan, and the cause was determined to be a change in their processes of tryptophan synthesis.^18^–^23^ The EMS outbreak prompted the United States Food and Drug Administration (FDA) to place a ban on all over-the-counter uses of tryptophan supplements, allowing only limited regulated use of tryptophan produced by United States manufacturers. Following the identification of the source of the outbreak, the ban was lifted in 2001.^23^ Since then, numerous research and clinical trials have been conducted without incident.^7^,^24^–^34^

The purpose of the following review is to provide an overview of tryptophan as an essential amino acid, with emphasis on tryptophan’s role in the synthesis of brain serotonin. The importance of tryptophan for a multitude of metabolic functions, and information on the research methodologies and uses, as well as therapeutic uses of tryptophan are discussed.

## Metabolic processes

### Protein synthesis

The principal role of tryptophan in the human body is as a constituent of protein synthesis. Because tryptophan is found in the lowest concentrations among the amino acids, it is relatively less available and is thought to play a rate-limiting role during protein synthesis.^3^,^5^ Tryptophan is also the precursor of two important metabolic pathways, kynurenine synthesis^5^,^12^,^13^ and serotonin synthesis.^5^,^7^,^35^–^37^

### Kynurenine synthesis

After protein synthesis, the second most prevalent metabolic pathway of tryptophan is for the synthesis of kynurenine, which accounts for approximately 90% of tryptophan catabolism.^5^,^12^, ^37^ Kynurenine is a key component in the synthesis of a number of metabolites, but most importantly, it is the precursor of kynurenic and quinolinic acids. Each of these metabolites has the potential to affect other neurotransmitters; specifically kynurenic acid is a glutamate receptor antagonist, while quinolinic acid is a glutamate receptor agonist.^39^ Among other pathways, kynurenine is known to be involved in acting as an ultra violet (UV) filter which protects the retina of the eye from UV damage.^40^,^41^ The effectiveness of this protection deteriorates with age, contributing to the normal changes in coloration and fluorescence of the lens that interfere with visual function and may, in some individuals, play a role in cataract formation.

### Serotonin synthesis

It is estimated that 95% of mammalian serotonin is found within the gastrointestinal tract,^42^ and only 3% of dietary tryptophan is used for serotonin synthesis throughout the body.^43^ Nevertheless, serotonin synthesis is one of the most important tryptophan pathways and a topic of intense research. It is estimated that only 1% of dietary tryptophan is used for serotonin synthesis in the brain,^12^,^44^ but despite the relatively low concentration of brain serotonin compared to that in the rest of the body, it has a broad impact as a neurotransmitter and neuromodulator and has been implicated in numerous psychiatric conditions and psychological processes.

### Tryptamine synthesis

In addition to tryptophan’s three major activities of protein, kynurenine, and serotonin synthesis, tryptamine is another biologically active compound that is derived from tryptophan. The immediate decarboxylation of tryptophan results in the synthesis of trace amounts of tryptamine (i.e. ng/g), which is an important neuromodulator of serotonin.^45^ Numerous animal studies have indicated that tryptamine acts as a control for the balance between excitatory and inhibitory functions of serotonin, and in other instances, tryptamine acts as a neurotransmitter with specific receptors that are independent of serotonin function.^45^

### Melatonin synthesis

Melatonin is a hormone produced in the tryptophan/serotonin pathway,^46^,^47^ which regulates diurnal rhythms and influences the reproductive and immune systems,^47^ as well as digestive processes and gastrointestinal motility.^48^ Melatonin synthesis is regulated by the blue light spectrum (i.e. 446 to 477 nm) in both artificial and sun light.^49^–^51^ During periods of darkness, it is actively secreted from the pineal gland to induce neural and endocrine effects that regulate circadian rhythms of behavior, physiology, and sleep patterns.^52^

### NAD/NADP synthesis

Tryptophan also plays a role as a substrate for synthesis of the coenzymes nicotinamide adenine dinucleotide (NAD) and NAD phosphate (NADP).^5^,^12^,^53^,^54^ NAD and NADP are coenzymes essential for electron transfer reactions (i.e. redox reactions) in all living cells. These enzymes can be synthesized de novo from ingested tryptophan, or from ingestion of niacin (i.e. vitamin B3).

### Niacin synthesis

Interestingly, tryptophan can act as a substrate for niacin synthesis^5^,^12^ through the kynurenine/quinolinic acid pathway. However, this is a less efficient use of tryptophan since approximately 60 mg of tryptophan are necessary to generate a single milligram of niacin.^5^,^11^,^55^ The recommended daily allowance of niacin is only 16 mg/day for men and 14 mg/day for women.^56^ For adults, in the United States, the median intake of niacin from food is approximately 41 mg/day for men and 28 mg/day for women,^57^ leaving little need for additional synthesis from tryptophan.

### Other metabolic functions

Tryptophan also exerts effects on other neurotransmitters and CNS compounds. Dopamine, norepinephrine, and beta-endorphin have been shown to increase following oral dosing of tryptophan. 42,58–61 Through serotonin synthesis, tryptophan is also thought to be involved in modulation of the endocrine system and cortisol,^60^,^62^ as well as prolactin and growth hormone.^63^,^64^

In summary, while tryptophan is found in the smallest concentrations of the 20 amino acids in the human body,^9^–^11^ it has wide-ranging effects and is a critical component of a multitude of essential metabolic functions. While there are three primary functions of tryptophan (i.e. protein, serotonin, and kynurenine synthesis), the focus of the remainder of this discussion is on tryptophan’s role in the synthesis of serotonin in the brain, and the utility of tryptophan for both research and clinical purposes.

### Pharmacokinetics

Tryptophan is the sole precursor of serotonin^35^ and, once consumed, tryptophan is distributed throughout the human body in the circulatory system. Unlike the other 19 amino acids, approximately 75% to 85%^3^,^65^ of circulating tryptophan is bound to albumin, with some estimates as high as 95%.^66^ It is primarily the non-bound, free tryptophan that is available for transport across the blood-brain barrier.^3^,^5^,^35^,^66^–^68^ However, since tryptophan has a higher affinity for the blood-brain barrier (BBB) transporter than it does for albumin,^3^–^69^,^70^ albumin-bound tryptophan that is in close proximity to the BBB will likely dissociate from the albumin to be taken up into the brain.^3^ Because of this difference in affinity, some researchers have concluded that up to 75% of albumin-bound tryptophan may be available to cross the blood-brain barrier.^3^ In the bloodstream, tryptophan competes with other large neutral amino acids (LNAA; e.g. histidine, isoleucine, leucine, methionine, phenylalanine, threonine, tyrosine, and valine) for the BBB transporter. 3,35,36,71,72 Given that BBB transporter is nearly saturated at normal plasma concentrations of amino acids, it is uniquely susceptible to competitive inhibition.^73^ Because of the competitive transport among the LNAAs, the bioavailability of tryptophan for transport across the BBB is best expressed by the ratio of tryptophan to the sum of its competing amino acids.^9^,^36^,^66^,^74^ Therefore, changing the ratio of tryptophan to the other competing large neutral amino acids can significantly affect concentrations of brain tryptophan available for serotonin synthesis. This can be accomplished by changing plasma concentrations of tryptophan, or by changing concentrations of the CAAs, either of which affect tryptophan availability and, by extension, serotonin synthesis.^75^–^78^ Although other influences, such as stress, insulin resistance, magnesium or vitamin B6 deficiency, and increasing age, can affect the rate of serotonin synthesis,^79^ fluctuations in the tryptophan/CAA ratio and changing tryptophan availability are the two factors most likely to affect this process.

To some extent, tryptophan availability to the brain can be enhanced by ingestion of carbohydrates and reduced by ingestion of proteins. Carbohydrate ingestion does not change the levels of circulating tryptophan, but it does decrease concentrations of CAAs through activation of insulin,^3^,^5^ which increases the relative availability of tryptophan for transport into the brain.^5^,^66^,^80^,^81^ In contrast, protein contains relatively low concentrations of tryptophan and ingestion of a protein meal increases the CAA concentration relative to tryptophan.^5^,^66^ The result is a larger percentage of circulating CAAs, which increases the competitive advantage over tryptophan for crossing the blood-brain barrier. This advantage is reflected in a smaller tryptophan/CAA ratio.^5^,^66^,^81^,^82^ Therefore, the ingestion of carbohydrates or proteins has the potential to change the availability of tryptophan for synthesis of brain serotonin; however, even small amounts of protein (as little at 4%) in a carbohydrate meal can prevent the increase in the tryptophan/CAA ratio.

The ability of carbohydrate and protein meals to modify tryptophan availability may be dependent on the time of ingestion.^83^,^84^ In one study, comparison of three calorically equivalent high carbohydrate meals across the day showed an elevated tryptophan/CAA ratio only after the first meal of the day.^83^ Another study^85^ showed that evening meals comprised of either 20% protein or 500 kcal carbohydrates had no significant effect on the tryptophan/CAA ratio. In contrast, a subsequent study administered a standard breakfast at 8 AM and a test lunch administered 4 hours later. Results showed that a “starch” (63% carbohydrates) or “sucrose” (73% carbohydrates) test lunch significantly increased the tryptophan/CAA ratio for the next 5 hours, while a lunch of protein (47% protein) significantly decreased the ratio for the next 4 hours.^82^ However, the former two studies used energy equivalent meals while the latter did not, which may be the source of the discrepant results. The breakfast in the latter study was comprised of 220 kcal, whereas the starch and sucrose test meals were 632 and 672 kcal (respectively) and the protein meal was 615 kcal. When taken together, the findings from these studies suggest that changes in tryptophan availability can be manipulated to some extent through dietary intake, although it is unlikely that ordinary changes in dietary tryptophan or the CAAs through protein or carbohydrate manipulations will produce changes substantial enough to have a noticeable impact on behavior in a healthy individual.^5^,^86^

In addition to these dietary factors that affect tryptophan’s availability for synthesis of brain serotonin, acute alcohol consumption has also been shown to decrease the tryptophan/CAA ratio by about 10% at about 30 minutes and 20%–25% at about 1.5 to 2 hours following ingestion.^87^,^88^ This decrease suggests that brain serotonin synthesis is impaired under these conditions.^87^,^89^ Where the average individual is likely to tolerate this level of serotonin depletion without undue effects on their behavior, vulnerable individuals may experience a larger depletion effect (e.g. 50% or more).^90^,^91^ This vulnerability may be due to a preexisting condition of low or borderline serotonin function that could be further impaired by the diminished serotonin synthesis following acute alcohol consumption.^88^,^89^

## Research Methodologies of Tryptophan Manipulation

While there are a number of methodologies used to study serotonin dysregulation, one of the most widely used methods is to reduce brain serotonin synthesis, typically by reduction of tryptophan availability. Experimental manipulations of tryptophan are dependent on the two-step process required for serotonin synthesis in the brain.^35^ First, brain tryptophan is converted to 5-hydroxytryptophan by the tryptophan hydroxylase enzyme (the rate-limiting step of serotonin synthesis). Second, 5-hydroxytryptophan is converted to serotonin by the aromatic amino acid decarboxylase enzyme. It is the activity of tryptophan hydroxylase that is dependent on the availability of brain tryptophan.^9^,^35^–^37^,^66^ Because tryptophan hydroxylase is typically 50% saturated with its tryptophan substrate, an increase or decrease of tryptophan availability in the brain can increase or decrease brain serotonin synthesis.^34^,^37^,^78^,^88^,^92^–^94^ Tryptophan hydroxylase is also dependent on oxygen^95^ and tetrahydrobiopterin^96^ as cofactors that regulate its enzymatic activity.

The ability to change the rates of serotonin synthesis is the foundation of a large body of research examining the relationship of serotonin dysregulation to mood, behavior, and cognition. One experimental method that has been used for studying the effects of decreased serotonin synthesis is the use of parachlorophenylalanine (PCPA).^66^ Also known as fenclonine, PCPA is a synthetic amino acid that is a selective inhibitor of tryptophan hydroxylase and found to cause a nearly complete and irreversible inactivation of tryptophan hydroxylase activity in studies of rat brain.^97^,^98^ However, in the rat, as PCPA is metabolized over time, the blockade is eventually reversed as new tryptophan hydroxylase is synthesized.^97^ Early studies of the relationship between serotonin and depression used this approach to block serotonin synthesis.^99^–^101^ While this method produced clear reductions in serotonin synthesis, it was not pursued in human research due to a number of negative side effects, including the potential for allergic eosinophilia.^66^ Another method for examining the effects of reduced serotonin synthesis is to experimentally restrict dietary intake of tryptophan, which slowly reduces tryptophan availability. However, this method is limited by a relatively lengthy period of dietary restrictions (e.g. up to 10 days), which have shown only 15% to 20% reductions of the plasma total tryptophan with minimal behavioral or neurochemical effects in humans.^5^,^66^,^86^

### Acute tryptophan depletion and loading

Much more pronounced reductions of plasma tryptophan can be obtained using the acute tryptophan depletion methodology, which produces maximal (but transient) tryptophan depletion within 5 to 6 hours. This method typically involves the administration of an amino-acid beverage that contains approximately 100 g of 15 amino acids (see Table 2), but lacks tryptophan.^66^,^77^,^102^ Consumption of this beverage results in two separate processes that reduce the availability of tryptophan for crossing the blood-brain barrier. First, the intake of the large amount of amino acids stimulates protein synthesis in the liver; however, without a proportionate intake of tryptophan, the protein synthesis clearly reduces the concentration of existing plasma tryptophan.^9^,^25^,^26^,^35^–^37^,^66^,^103^ Second, the small amount of plasma tryptophan relative to the high concentration of plasma CAAs further decreases the availability of tryptophan for crossing the blood-brain barrier. Both ongoing protein synthesis and a lower plasma tryptophan/CAA ratio maximize the competitive disadvantage for tryptophan transport into the brain.^36^,^77^ This two-fold effect results in a significant decrease of brain serotonin synthesis in both human and non-human primates,^77^,^78^,^104^,^105^ and studies of rat brain have also shown reductions of neuronal serotonin release.^66^,^106^,^107^ Conversely, serotonin synthesis can be increased using acute tryptophan loading, which is often used as a control condition for depletion. Tryptophan loading is accomplished by adding a disproportionately large amount of tryptophan to the amino-acid formulation. This large amount of tryptophan maximizes tryptophan’s competitive advantage and increases the availability of tryptophan for brain serotonin synthesis.^37^ The specificity of tryptophan depletion appears to be established by comparing this formulation with an alternative formulation designed to deplete lysine (an arbitrarily chosen essential amino acid). Mood and memory effects were specific to tryptophan depletion, which would seem to rule out general inhibition of protein synthesis that would also likely impair mood and memory functions. Moreover, when compared to control conditions (e.g. tryptophan loading or a balanced formulation), effects are specific to the depletion.^32^,^37^,^108^–^111^

### Effectiveness of tryptophan manipulations

Using both tryptophan depletion and loading, many studies have provided measures of the effectiveness of these manipulations for changing plasma tryptophan. A comparison across studies showed an 81% average reduction of plasma tryptophan following consumption of the most commonly used 100 g depletion formulation,^112^ with reductions ranging from 55% to 94%.^27^,^37^,^77^,^88^,^102^,^104^,^112^–^117^ Comparable results have been found following administration of a 50 g (i.e. half-size) formulation. For example, relative to pre-drink measures, two time-course studies showed robust depletions of 87% (i.e. free and total tryptophan;)^112^ and 89% (i.e. free tryptophan/CAA ratio;)^37^ maximal reduction of plasma tryptophan following consumption of the 50 g depletion formulation. Likewise, the 50 g and 100 g tryptophan loading formulations have also shown similar results, both of which produce marked increases in plasma tryptophan that range from 300% to 500% of pre-drink measures.^37^

A potential limitation of this methodology is that reductions of serotonin synthesis may not be uniform across all brain areas and may not be representative of neuronal release. However, positron emission tomography (PET) following tryptophan depletion showed reductions of serotonin synthesis were similar across multiple areas of the brain in spite of differences in the density of innervations,^78^ although specific areas affected may vary by sex.^118^ Furthermore, results from several rodent studies have provided supporting evidence that neuronal release of serotonin occurs in direct relationship to the concentration of its tryptophan substrate,^119^–^122^ however one rodent study indicated that physiological variables other than substrate availability may be of greater importance for regulating serotonin synthesis and release.^123^,^124^ Similarly, a recent rodent study examined the relationship between dietary tryptophan depletion and concentrations of extracellular serotonin. Plasma tryptophan was depleted by 70%, but microdialysis results showed no corresponding reductions of extracellular serotonin.^125^

### Methodological considerations

When designing research protocols to investigate various mood, behavior, or cognitive effects of this methodology, it is important to consider that the onset and duration of the peak change in plasma indicators of brain serotonin synthesis likely do not coincide with serotonergic changes in the brain, which may affect the experimental design. However, it is possible to infer both the onset and duration of significant changes in brain serotonin concentrations and function following tryptophan manipulations from studies that have concurrently measured both plasma tryptophan and central indicators (e.g. lumbar punctures measuring cerebrospinal fluid tryptophan or the primary metabolite of serotonin, 5-hydroxyindoleacetic acid) of changes in serotonin catabolism. Two previous studies have shown that these central measures reached their lowest point approximately two hours after the onset of the maximal changes of plasma tryptophan measures for both the 50 g^112^ and 100 g^104^ depletion formulations, and noted that this extended estimate does not account for time between brain changes and measurements taken at the lower end of the spinal column.^112^ Furthermore, a PET scan of the human brain indicated significant changes in serotonin synthesis occurred at 5 hours following amino acid consumption.^78^ Depending on the size of the drink (e.g. 50 g or 100 g) and the plasma measure used (e.g. total tryptophan or free tryptophan to competing amino acids), near maximal reductions of plasma measures remain for another 4 to 5 hours following onset (i.e. up to 8 to 10 hours following administration),^37^,^104^,^112^ which provides sufficient overlap for an optimal testing window at 6 to 7 hours following amino acid consumption.

Relative to the other methods for experimentally reducing brain serotonin synthesis (i.e. enzyme inhibitors and dietary manipulations), using acute tryptophan depletion and loading amino-acid formulations presents some significant advantages. These amino-acid manipulations are economical, safe, and minimally invasive, as well as highly effective for rapidly producing substantial changes in tryptophan availability to the brain. These effects are also transient and quickly reversed by returning to a normal diet.^126^ Furthermore, manipulating the underlying biology prior to testing also provides for interpretations of cause and effect relationships. For these reasons, acute tryptophan depletion has remained an important and popular research tool for understanding serotonergic dysregulation.

## Research Applications of Acute Tryptophan Depletion

Acute changes in tryptophan availability have been used to test a wide variety of basic psychological, behavioral, and physical processes,^66^,^127^ including: motion sickness,^128^ sleep,^129^,^130^ mood,^27^,^77^ visual discrimination,^30^ cognition,^34^ social information processing,^33^ and memory processes.^31^,^32^ This method has also been applied in investigations of numerous psychiatric disorders, including: Major Depressive Disorder,^131^ Seasonal Affective Disorder,^132^,^133^ Bipolar Disorder,^134^,^135^ Obsessive-Compulsive Disorder,^136^ Schizophrenia,^137^ Bulimia Nervosa,^115^ Premenstrual Syndrome,^138^ and Panic Disorder.^139^ While this is a widely-used technique, the majority of this research has been more specifically focused on mood and depression, memory and other cognitive functions, and behavior.

### Mood and depression

One of the earliest and most common uses of tryptophan depletion was for the study of changes in mood which are commonly believed to be related to serotonergic mechanisms. Some of the earliest studies found modest mood-lowering effects following acute tryptophan depletion in samples of healthy young men.^102^ Since then, results have varied considerably, such that approximately half of published studies have found no effects on mood in healthy adult samples.^102^,^140^ A number of these studies have reported that healthy women may be more vulnerable to the mood-lowering effects of tryptophan depletion than men,^138^,^113^,^141^,^142^ which is supported by imaging studies that provide evidence of sex differences in brain serotonin synthesis.^78^,^118^ The differences found between men and women are also consistent with the general consensus that conflicting findings appear to be the result of characteristic differences in the individuals being tested.^103^,^126^ For example, positive results have involved individuals with baseline depression scores at the upper end of normal, while negative results have generally been found in those with lower baseline scores.^126^,^143^,^144^ The lack of effect on mood following tryptophan depletion in rigorously screened healthy individuals (e.g. low baseline depression, aggression, or impulsivity scores) is supported by a similar lack of effect found in imaging studies (e.g. fMRI, PET).^145^

The extent of the effects of acute tryptophan depletion on mood appears to be related to varying levels of vulnerability to disturbance of the central serotonin system. Relative to healthy controls, there is more consistency of mood-lowering effects in healthy adults who may be vulnerable to serotonin disturbances, such as those with family histories of mood disorders^16^,^32^,^135^,^146^,^147^ or other underlying biological vulnerabilities (e.g. genetic or brain abnormalities).^148^,^149^ The most consistent effects on transient changes in mood states have been found in patients with remitted depression who are concurrently receiving antidepressant treatment. In 8 out of 10 studies, tryptophan depletion produced a temporary return of clinical symptoms in patients who were responsive to their treatments.^126^ Two prospective studies have also reported that the tryptophan depletion methodology may be a useful predictor of future depressive episodes. These studies administered tryptophan depletion to symptom-free, treatment-free individuals with a history of either a major depressive episode^150^ or seasonal affective disorder,^132^ and followed the individuals for up to one year. Results from both studies indicated that the individuals who responded with depressive symptoms during tryptophan depletion were at greater risk for subsequent depressive episodes than non-responders. In summary, these results suggest that serotonin dysfunction is a trait abnormality in depressive disorders,^144^ and that individuals with a particular biological vulnerability for future depression may be especially sensitive to even transient changes in serotonin availability.^150^

### Cognitive processes

In contrast to the typical lack of mood changes in healthy adults, tryptophan depletion has been demonstrated to affect a variety of cognitive processes in both healthy individuals and those with a serotonergic vulnerability. Impairments in a variety of learning and memory skills following tryptophan depletion are well documented. The most reliable findings are impairments of declarative episodic memory processes of delayed recall and memory consolidation.^151^ For example, in healthy adults, when a word list was learned at 6 hours after tryptophan depletion (i.e. during peak effect) and active recall was tested 30 minutes later, both recall and word recognition were impaired; however, when tested immediately following presentation of the word list, no effect was found.^152^ Riedel and colleagues concluded that compromised serotonergic activity impaired consolidation of information into long-term memory without any effect on short-term memory. These long-term memory deficits in delayed recall have been replicated in numerous studies using a variety of presentations, including visual and auditory presentation of words, as well as presentation of pictures and abstract shapes (for detailed reviews, see^151^,^153^). These effects have been found in healthy adult volunteers,^31^,^154^–^156^ in adults with a family history of bipolar disorder,^157^ and in clinical samples.^158^,^159^ For instance, in a comparison of adults with and without family histories of bipolar disorder, tryptophan depletion impaired long-term memory consolidation in both groups, and problem solving was also impaired in those with a family history, while problem solving improved in those without a family history.^32^

Tryptophan depletion has also been shown to impair learning on visual discrimination and memory retrieval,^114^ episodic memory,^155^ stimulus-reward learning,^30^ and cognitive flexibility,^160^ among other cognitive processes, although more studies are needed to test the reliability of these results. In an editorial commentary on cognitive effects of tryptophan depletion, Riedel^161^ notes that there are a number of other physiological effects that may result from tryptophan manipulations that could be involved in the modulation of cognitive functions, such as quinolinic acid (NMDA agonist), and kynurenic acid (NMDA, nicotinic, and glutamatergic antagonist), which should also be considered for measurement.^42^

### Behavior

Finally, tryptophan manipulations have a long history of studying behavior using laboratory-measures to assess social behavior and changes in aggression and impulsivity that may be dependent, in part, on changes of serotonin synthesis.^30^,^36^,^88^,^162^–^166^ For example, laboratory-measured aggression (i.e. Point Subtraction Aggression Paradigm, PSAP;)^110^,^111^ was shown to increase following tryptophan depletion, and this effect was greater in those that responded more aggressively before the manipulation.^111^ Additionally, following tryptophan depletion, men who reported high-trait aggression have shown increased laboratory aggressive behavior relative to low-trait aggressive men.^108^ Interestingly, among women, tryptophan depletion increased laboratory-measured aggression (i.e. PSAP) while tryptophan loading decreased aggression and this effect was specific to those women with elevated plasma tryptophan at baseline.^36^ This finding was supported by a study of healthy adult men and women who completed a number of measures of self-reported anger, hostility, and aggression that were related to their endogenous plasma tryptophan levels.^167^ Results showed that higher tryptophan levels were associated with elevated anger, hostility, and aggression scores in women, but not men. As noted in the previous study, this association was specific to women with elevated plasma tryptophan, compared to women with lower plasma tryptophan levels who were more agreeable, less hostile, and less likely to express their anger.

Both animal and human studies have shown that serotonin function is involved in inhibitory control of aggression.^168^–^170^ While reduced serotonergic functioning has been clearly associated with aggressive and violent behavior in general,^171^,^172^ it is more specifically related to impulsive aggression^168^,^170^ and likely to be involved in modulating inhibitory behavior and expression of impulsivity.^171^,^172^ This was demonstrated in a study of aggressive adolescents with Attention Deficit Hyperactivity Disorder (ADHD) where laboratory-measured impulsive aggression was increased following tryptophan depletion relative to a balanced control, and this effect was independent of age and intensity of ADHD symptoms.^173^ Similarly, in a sample of aggressive adolescent males, impulsivity was elevated compared to nonaggressives, but this elevation was the same with and without tryptophan depletion,^172^ although the authors suggest this was most likely due to a ceiling effect . In another study of young men with and without a family history of paternal alcoholism, tryptophan depletion showed no effect on aggressive responses during a modified Taylor aggression task in either group. However, increased disinhibition (on a go/no-go task) was demonstrated by the men with a family history of alcoholism relative to both placebo and men without a family history.^164^ These authors concluded that there are subsets of individuals who appear to be more vulnerable to serotonergic dysregulation and impulsive behavior. A recent examination of whether serotonin modulates impulsive behavior through mechanisms involved in emotion used tryptophan depletion and placebo control to test a laboratory model of self-regulation.^166^ Contrary to other results, reduced serotonin function increased the retaliation to perceived unfairness without changing response inhibition, mood, or reward processing. These divergent findings may be the result of methodological differences of testing paradigms that examine different underlying mechanisms,^174^ and/or these results may represent differences among the volunteer testing samples. Future studies that use multiple behavioral measures in the same experimental sample may clarify these conflicting findings.^174^

When examining the relationship of trait impulsivity to changes following tryptophan depletion, boys with ADHD were divided into high and low trait impulsivity groups. Using a competitive reaction time test, tryptophan depletion increased impulsive aggression of the low impulsive group, but not the high impulsive group.^175^ The authors suggested that the lack of effect in the high impulsive group was likely due to the difficulty of increasing already high rates of impulsivity that could not be further influenced by reduced serotonin, whereas the depletion effect could make the low impulsive group react as if they were high impulsive. Additionally, using a continuous performance test in normal healthy men, tryptophan depletion produced increased laboratory-measured impulsivity compared to placebo.^176^ Other studies, using stop-signal tasks have failed to find increased impulsive responding after tryptophan depletion.^177^,^178^ Using a stop-signal task, another group tested behavioral inhibition and learning using a selective serotonin reuptake inhibitor and a selective noradrenaline reuptake inhibitor, both known to rapidly increase brain serotonin and noradrenalin (respectively) in animal testing.^179^ Results indicated that increased noradrenaline improved inhibitory responding, but serotonin had no effect. Rather, increased serotonin impaired learning, whereas noradrenaline had no effect. This difference between continuous performance and stop-signal tasks may signify different underlying behavioral mechanisms governing these responses.

A recent review of brain activation in imaging studies (i.e. fMRI, PET)^179^ examined results from studies using a variety of cognitive tasks (e.g. response inhibition, learning, response interference, verbal fluency). The authors concluded that overall, results appear to indicate involvement of serotonin dysregulation in cognitive impairments, but the number of divergent results remains puzzling. Contrary findings from different studies may be the result of a variety of explanations and a host of methodological differences, including use of tasks that measure different underlying processes and testing samples of individuals who differ in personality, gender, family histories, and genetic vulnerabilities. While serotonin plays a part in cognitive functions, inconsistencies across studies need to be addressed in the future both to control for, and study, interindividual differences.

## Therapeutic Uses of Tryptophan

While dietary intake alone (i.e. ingestion of food) would seldom influence the availability of tryptophan significantly, administration of exogenous tryptophan has been the focus of numerous clinical research and homeopathic applications. One of the earliest examples was an attempt by Lauer and colleagues^180^ to augment treatment response for schizophrenia by combining tryptophan administration with iproniazid, a monoamine oxidase inhibitor (MAOI). The success of the combined treatment compared to the MAOI alone changed how researchers thought about the influence of tryptophan treatment on brain function,^84^ and began a series of clinical trials testing the efficacy of treatment for a number of clinical disorders that yielded promising but often inconclusive results. Tryptophan has been used for a broad spectrum of clinical applications, such as treatment of pain, insomnia, depression, seasonal affective disorder, bulimia, premenstrual dysphoric disorder, attention deficit/ hyperactivity disorder, and chronic fatigue (see^17^,^84^). Tryptophan has also been widely used as an over-the-counter, natural remedy for depression, pain, insomnia, hyperactivity, and eating disorders.^17^

The therapeutic use of tryptophan for treatment of clinical disorders and syndromes has concentrated primarily on increasing tryptophan intake for the treatment of depressive disorders and related conditions, although other psychiatric and medical conditions appear to be somewhat responsive to tryptophan treatment. One of the most frequent clinical uses of tryptophan has been for the treatment of major depression; however, clinical findings of the efficacy of tryptophan treatments are mixed.

### Depression

Tryptophan has been found to be as effective as tricyclic antidepressants in a number of trials,^181^–^183^ and one study found that the effects of tryptophan and amitriptyline, alone and in combination, were all superior to placebo.^184^ However, other studies with tricyclic antidepressants have shown inconsistent efficacy for treating depressive symptoms.^184^–^187^

Studies of tryptophan in combination with electroconvulsive therapy (ECT) have also produced inconsistent findings. One study demonstrated that patients with depression who received combined doses of tryptophan (3 g/day) and nicotinamide for 4 weeks reported significantly lower ratings of depression compared to those who received ECT twice weekly.^188^ Conversely, in another study, patients with a severe primary depressive illness treated with ECT improved significantly compared to those receiving tryptophan (6 to 8 g) plus pyridoxine daily.^189^ Similarly, patients receiving ECT twice daily improved significantly compared to patients receiving daily doses of combined tryptophan (6 g) and pyridoxine.^190^ In yet another study, there were no significant differences in depressed patients treated with tryptophan alone (6 g/day) compared to ECT alone.^191^

In contrast to the mixed results of the effects of tryptophan with tricyclic antidepressants or ECT, tryptophan has been shown to be more effective in combination with monoamine oxidase inhibitors (MAOI). For example, depressed patients who were unresponsive to a 60 mg/day dose of the MAOI phenelzine received supplements of 12, 15, or 18 g of tryptophan or placebo. A significantly higher percentage of the patients on the combined therapy (i.e. MAOI plus tryptophan) improved compared to those receiving the MAOI alone or with placebo.^192^ Patients who received a combined therapy of tryptophan (6 g/day) and a different MAOI, nialamide, also improved significantly compared to those who received nialamide alone.^185^ In another study, compared to patients who received doses of the MAOI tranylcypromine plus placebo for one week, those who received a 214 mg/kg/day supplement of tryptophan reported a significant decrease in depression ratings during that week, as well as during the 2 weeks after tryptophan supplements were discontinued.^193^

Although the results of the therapeutic combination of tryptophan with MAOIs have demonstrated the most successful results for treatment of depression, most clinical studies have produced mixed results as to the efficacy of tryptophan for treatment of depression. These mixed results are due, in part, to flawed study designs and trials using insufficient lengths of time to allow determination of efficacy. Methodological differences such as inconsistent diagnostics within and across studies^43^ have also produced mixed results. Taken together, there is evidence that tryptophan is effective for ameliorating mild to moderate depressive symptoms, but not severe depressive symptoms.^84^

### Other mood disorders

Tryptophan has been used successfully in the treatment of seasonal affective disorder and may be as effective as light therapy. In one open-label study, 16 patients who met DSM-IV criteria for a recurrent major depressive disorder with a seasonal (winter) pattern were treated with light therapy for 2 weeks. The treatment for those who were partial or non-responders to this light therapy was then augmented with tryptophan (3 g/day) for 2 weeks, which produced a marked response to treatment.^28^ In a second study, patients who met criteria for major depression with a seasonal pattern were treated with combined light therapy and tryptophan. Half received 2 weeks of light therapy first and the other half received 4 weeks of tryptophan treatment first (with a 1-week washout between treatments). While one third of the patients showed no response to either treatment, over half of the patients showed significant improvement during both treatments regardless of the order of treatment.^29^

Steinberg and colleagues conducted a randomized, double-blind, placebo-controlled trial to assess the efficacy of tryptophan (6 g/day) for treating Premenstrual Dysphoric Disorder symptoms.^194^ Those patients receiving tryptophan reported significant reductions in dysphoria, mood swings, and irritability compared with those receiving placebo. These effects were thought to be the result of increased kynurenine synthesis during the late-luteal phase of the menstrual cycle.^195^

### Sleep disorders

Tryptophan has also been used for the treatment of sleep disorders, and is thought to produce its therapeutic effects through melatonin mechanisms. Improvement in sleep latency has been reported^196^,^197^ with doses as low as 1 g,^198^ and improved Stage IV sleep has been reported with doses as low as 250 mg.^198^ An important feature of tryptophan treatment is that, unlike many other medications administered for sleep disorders, it does not limit cognitive performance or inhibit arousal from sleep.^197^,^199^ Tryptophan also produces significant improvements in obstructive sleep apnea, but not central sleep apnea.^200^ After an average dose of 2.5 mg of tryptophan administered at bedtime, patients with obstructive sleep apnea showed significant improvement while those with central sleep apnea did not.

### Other uses

In patients undergoing smoking cessation, tryptophan (50 mg/kg/day) has been used in combination with a high carbohydrate diet, and is reported to reduce anxiety and severity of withdrawal symptoms, and to improve abstinence or reduce the number of cigarettes smoked.^201^ However, tryptophan treatment has been reported to have no effect on bruxism^202^ and, in combination with dietary manipulations, tryptophan treatment has also shown no effect on chronic myofascial pain.^203^

## Conclusions

In summary, tryptophan is a unique amino acid that is an essential component of the human diet. Although it has the lowest concentration in the human body relative to the other 19 primary amino acids, tryptophan is a critical component of numerous metabolic functions. Despite the side effects noted above and past concerns about the safety of tryptophan as a treatment or nutritional supplement, tryptophan has been widely used in numerous research and clinical trials without incident for nearly 25 years e.g.^9^,^32^,^36^,^37^,^64^,^66^,^88^,^103^,^111^,^131^,^141^,^195^,^204^ Experimental research has shown that tryptophan can be an important determinant of mood, cognition, and behavior. Although results have been inconsistent, clinical trials have provided some initial evidence of tryptophan’s efficacy for treatment of psychiatric disorders, particularly when used in combination with other therapeutic agents.

To improve the utility of tryptophan research for understanding the relationship of serotonin dysregulation as an underlying mechanism in psychiatric disorders, as well as behavioral, cognitive, and physical problems, it will be important to understand the factors that have contributed to the inconsistent results from previous studies. To advance the efficacy and utility of tryptophan for therapeutic purposes, future clinical studies will need to improve on methodological pitfalls made in the past. Such considerations would include employing systematic control of dosing, standardization of both research and treatment methodologies, and improved diagnostics of psychiatric disorders to test more homogeneous groups of patients, as well as careful selection and matching of patient and control groups (or conditions) being tested.

## Figures and Tables

**Table 1.** The *L*-tryptophan and competing amino acids (CAAs) found in common foods. The *L*-tryptophan/CAA ratio represents the relative availability of plasma *L*-tryptophan for crossing the blood-brain barrier and is thought to be the best indicator of brain serotonin synthesis.

	*L*-tryptophan*(mg)	Sum of CAAs** (mg)	**Ratio**
Turkey, Skinless, Boneless, Light Meat (per pound, raw)	410	9,525	0.043
Chicken, Skinless, Boneless, Light Meat (per pound, raw)	238	5,122	0.046
Turkey, Skinless, Boneless, Dark Meat (per pound, raw)	303	7,036	0.043
Chicken, Skinless, Boneless, Dark Meat (per pound, raw)	256	5,492	0.047
Whole Milk (per quart)	732	8,989	0.081
2% Milk (per quart)	551	12,516	0.044
Wheat Bread (per slice)	19	317	0.060
White Bread (per slice)	22	439	0.050
Semisweet Chocolate (per ounce)	18	294	0.061
Sweet Chocolate (per ounce)	16	270	0.059
Canned Tuna (per ounce)	472	10,591	0.045
Cheddar Cheese (per ounce)	91	2,298	0.040
Peanuts (per ounce)	65	1,574	0.041
Oats for Oatmeal (per cup)	147	2,617	0.056
Dried Prune (one)	2	27	0.074
Banana (one medium)	11	237	0.046
Apple (one medium)	2	70	0.029

* e.g. The recommended daily allowance for a 79 kg (175 lb) adult is 278 to 476 mg.

** CAAs = Isoleucine, Leucine, Phenylalanine, Tyrosine, and Valine, the five large neutral amino acids typically included in the tryptophan/CAA ratio.

**Table 2.** Amino acid compositions of 50 g and 100 g *L*-tryptophan depletion and loading formulations.

***L*-tryptophan Formulation**	**50 g**	**100 g**
*L*-tryptophan Depletion	0.00	0.00
*^‡^°*L*-tryptophan Loading	5.15	10.30
15 Amino Acids		
*L*-alanine	2.75	5.50
*L*-arginine	2.45	4.90
*L-cysteine	1.35	2.70
Glycine hydromonochloride	1.60	3.20
*L-histidine	1.60	3.20
*^‡^°*L*-isoleucine	4.00	8.00
*^‡^°*L*-leucine	6.75	13.50
°*L*-lysine	4.45	8.90
*°*L*-methionine	1.50	3.00
*^‡^°*L*-phenylalanine	2.85	5.70
*L*-proline	6.10	12.20
*L*-serine	3.45	6.90
*°*L*-threonine	3.25	6.50
*^‡^°*L*-tyrosine	3.45	6.90
*^‡^°*L*-valine	4.45	8.90
Depletion, total grams	50.00	100.00
Loading, total grams	55.15	110.30

* Competing amino acids (CAA);

‡ CAAs typically summed for calculating the tryptophan/CAA ratio;

° Essential amino acids.
